# Hyaluronic Acid Promotes the Osteogenesis of BMP-2 in an Absorbable Collagen Sponge

**DOI:** 10.3390/polym9080339

**Published:** 2017-08-04

**Authors:** Hairong Huang, Jianying Feng, Daniel Wismeijer, Gang Wu, Ernst B. Hunziker

**Affiliations:** 1 Department of Oral Implantology and Prosthetic Dentistry, Academic Centre for Dentistry Amsterdam (ACTA), Universiteit van Amsterdam and Vrije Universiteit Amsterdam, Gustav Mahlerlaan 3004, 1081LA Amsterdam, Nord-Holland, The Netherlands; hhrstudy@126.com (H.H.); d.wismeijer@acta.nl (D.W.); 2 School of Stomatology, Zhejiang Chinese Medical University, Hangzhou 310053, China; twohorsejy@163.com; 3 Departments of Osteoporosis and Orthopaedic Surgery, Inselspital (DKF), University of Bern, Murtenstrasse 35, 3008 Bern, Switzerland; ernst.hunziker@dkf.unibe.ch

**Keywords:** hyaluronic acid, bone morphogenetic protein-2, absorbable collagen sponge, bone regeneration, angiogenesis

## Abstract

(1) Background: We tested the hypothesis that hyaluronic acid (HA) can significantly promote the osteogenic potential of BMP-2/ACS (absorbable collagen sponge), an efficacious product to heal large oral bone defects, thereby allowing its use at lower dosages and, thus, reducing its side-effects due to the unphysiologically-high doses of BMP-2; (2) Methods: In a subcutaneous bone induction model in rats, we first sorted out the optimal HA-polymer size and concentration with micro CT. Thereafter, we histomorphometrically quantified the effect of HA on new bone formation, total construct volume, and densities of blood vessels and macrophages in ACS with 5, 10, and 20 μg of BMP-2; (3) Results: The screening experiments revealed that the 100 µg/mL HA polymer of 48 kDa molecular weight could yield the highest new bone formation. Eighteen days post-surgery, HA could significantly enhance the total volume of newly-formed bone by approximately 100%, and also the total construct volume in the 10 μg BMP-2 group. HA could also significantly enhance the numerical area density of blood vessels in 5 μg BMP-2 and 10 μg BMP-2 groups. HA did not influence the numerical density of macrophages; and (4) Conclusions: An optimal combined administration of HA could significantly promote osteogenic and angiogenic activity of BMP-2/ACS, thus potentially minimizing its potential side-effects.

## 1. Introduction

Recombinant human bone morphogenetic protein-2 (BMP-2) has been in clinical use mainly for the generation of spinal fusions for more than a decade [1,2]. In recent years, BMP-2 has also been proven to be an efficacious way to promote bone regeneration in the field of dentistry and maxillofacial surgery, such as ridge aµgmentation [3], sinus lift [4], and periodontal and peri-implant [5] bone regeneration. It is able to accelerate bony healing processes, and substitute autologous bone transplantation [6,7]. Overall, its clinical use is quite successful; however, the use of BMP-2 is, unfortunately, associated with a number of severe undesired side effects that are able to seriously impair the health of patients and the musculoskeletal functions of the treated patients [7,8]. Such side-effects include, among others, ectopic bone formation, paralysis, and neurological disturbances [9,10]; but malignant pathologies are not involved [11,12].

BMP-2 is clinically applied topically in a free form together with an absorbable collagenous sponge (ACS) [13]. The recommended dose is exceedingly high (12 mg/ACS unit; i.e., approximately 37.3 mg of BMP-2 per gram of ACS sponge); and in this high dosage scheme probably lies the reason for many of the untoward side effects [6,9]. It is, however, not only the dosage that is able to influence the response of the targeted populations of progenitor cells and their differentiation pathways, but also the mode of application and the manner in which the agent is locally presented to the targeted cell populations. On the other hand, the microenvironment (niche conditions) in which the desired bone formation activity is aimed to take place also has a significant influence on the degree and speed of the process, as well as the type of ossification process (enchondral or desmal); for example, the local biomechanical niche conditions are able to influence this process [14], but less so with respect to the density of blood vessels present [15], even though the high numbers of blood vessels establish the presence of large numbers of perivascular adult stem cells [16] as a source of precursor cells for osteogenesis [17]. For this reason some researchers described previously [18] that a sequential release of an angiogenic factor (initial release) with the osteogenic factor (BMP-2; delayed release) is able to accelerate bone formation activities.

Respecting the methods of enhancement of BMP-2 bioactivity, glycosaminoglycans (GAGs) have been described previously to have such a potential, in particular relating to the desired osteogenesis effects [19]. Hyaluronic acid (HA) belongs, chemically, to the large groups of GAGs [20]; they are a group of large linear polysaccharides constructed of repeating disaccharide units, containing amino sugars and uronic acid, and are one of the most frequently-used tools to improve the microenvironment for BMP-induced osteogenesis. It has been found that the active components in GAGs for this desired osteogenic enhancement effects are able to bind, stabilize and present growth factors to cells for improved receptor interaction [21]. Furthermore, they can direct the immediate signaling activities of BMP2 throµgh enhancing the subsequent recruitment of type II receptor subunits to BMP-type I receptor complexes [22]. As one of the main GAG components, HA can be a promising drug to promote the osteogenic potential of BMP-2. HA is able to stimulate osteoinduction activities in bone wound healing processes [19]. In particular, high-molecular weight HA (≈1900 KDa) was found in animal experiments to be able to promote this effect. Huang et al. [23] found that low molecular weight HA (60 kDa) and high-weight HA (900 and 2300 kDa) were able to significantly stimulate cell growth and to increase osteocalcin mRNA expression levels. In addition, it was revealed in previous research that HA is involved in several biological processes [24], such as cell differentiation [25], angiogenesis [26], morphogenesis [27], and wound healing [28]; furthermore, HA was described to be able to inhibit osteoclast differentiation [29] in addition to its downregulation potential of BMP-2 antagonists [30].

In this study we hypothesize that a combination use of BMP-2 with HA is able to promote the osteogenesis activity in a subcutaneous bone induction model at lower dosage levels of BMP-2 in ACS.

## 2. Materials and Methods 

### 2.1. Experimental Design

We proceeded in two steps: initially, we performed screening experiments in a subcutaneous bone induction model to determine the optimal HA polymer size and concentration to be used for the main experiment. In the main experiment we elucidated the optimal dosage of BMP-2 to be used together with ACS and HA within a time period of 18 days.

### 2.2. Animals, Anesthesia, and Surgery

The animal experiment was approved by Ethical Committee of School of Stomatology, Zhejiang Chinese Medical University. All animal experiments were carried out according to the ethics laws and regulations of China and the guidelines of animal care established by Zhejiang Chinese Medical University. (Sprague-Dawly) SD rats (mean weight: 230 g, range from 190 to 250 g) were used in this study for all experiments. The animal experiments, such as anesthesia, sample randomization, and surgery were performed as we previous described [15].

### 2.3. Screening Experiments

The HA screening experiments were performed using five different HA polymer lengths to be tested, and each one of them was tested at 6 different concentrations of the polymer, and at three different dosages of BMP-2 (see Table 1).

Each of the HA polymer test was performed in the presence of ACS (Inductos^®^, Medtronic, Minneapolis, MN, USA) (identical circular ACS samples were prepared of 8 mm diameter), and with 5, 10, or 20 µg of BMP-2 (Inductos^®^, Medtronic, Minneapolis, MN, USA). BMP-2 portions were added to ACS sponges from syringes; thereafter, the HA-solution was added (20 µL portions per sample), just before implantation. The choice of three different dosages of BMP-2 was determined according to previous publications [31,32]. In these screening experiments one test sample was implanted in 35 SD rats on the left and right back side per animal. The evaluations of the degrees of osteoinduction obtained were performed using micro CT scans (Skyscan1176, Bruker, Kontich, Belgium) and the results were assessed by two independent observers for maximum subcutaneous bone signal intensity.

### 2.4. Main Experiment

Twenty-four eight-week-old male SD rats were used for the main experiment; and in each animal two 8 mm diameter BMP-2/ACS implants were placed. Eight experimental groups (*n* = 6 samples and six animals per group) were set up as following:

G1: no BMP-2, ACS alone;
G2: BMP-2/ACS, 5 µg BMP-2;
G3: BMP-2/ACS, 10 µg BMP-2;
G4: BMP-2/ACS, 20 µg BMP-2;
G5: no BMP-2, ACS alone + 2µg HA;
G6: BMP-2/ACS (5 µg BMP-2) + 2 µg HA;
G7: BMP-2/ACS (10 µg BMP-2) + 2 µg HA;
G8: BMP-2/ACS (20 µg BMP-2) + 2 µg HA.

A preimplantation control group of ACS sponges was also included in the study in order to determine the basic carrier volume before implantation as a time 0 reference volume.

In the groups containing HA, this compound was used at a concentration of 100 µg HA/mL, and the amount of 20 µL solution was added per sample. Samples were then stored overnight under aseptic conditions in a sterile hood for induction of sample drying before implantation.

### 2.5. Tissue Processing

Eighteen days post-operation the implanted samples were retrieved together with the surrounding tissues and chemically fixed, dehydrated, and embedded in methylmethacrylate; sections of 600 µm in thickness were produced and taken with a 1000 µm-interval between two adjacent sections. The sections were thereafter glued to Plexiglas boards, polished down (sand paper) to 100 µm thickness, and then stained with McNeal's tetrachrome, toluidine blue O, and basic fuchsin, as described previously [15].

### 2.6. Histomorphometry and Stereology

The histological sections were photographed at a final magnification of 200× under an Eclipse 50i light microscope (Nikon, Tokyo, Japan), and photographic subsampling was performed according to a systematic random-sampling protocol [33]. Using the photographic prints, the areas of the implants and the areas of newly-formed bone tissue were measured histomorophometrically using point counting methods [33]. Mineralized bone tissue (stained pink) and unmineralized bone tissue (light blue) (see Light micrographs of BMP-2/ACS constructs) were defined as newly-formed bone tissue; areas of collagen carrier material were measured the same way [34].

### 2.7. Stereological Estimators

Volume Estimators. The preimplantation reference volumes of the collagenous carrier materials (*n* = 6) were estimated using the principle of Cavalieri [35], as well as the final remaining total tissue volumes [33] at the end of the implantation time period (18 days). The degree of carrier degradation was computed by dividing the reference volume of carrier material at time point zero divided by the carrier material volume present at the end of the experiment. The areas of newly-formed bone tissue and remaining carrier materials were estimated at final magnifications of 200×, and were subsampled according to a systematic random protocol [33,35]. 

Numerical Estimators. Blood vessel area density and blood vessel numerical area density (number of blood vessel cross-sections per unit tissue area) (at 200× magnification) as well as macrophage numerical area densities (at 400× magnification) were estimated as previously described [33].

### 2.8. Statistical Analysis

All data are presented as mean values together with the standard error (SE) of the mean. Differences between the experimental groups were analyzed using the one-way ANOVA-test. Statistical significance was defined as *p* < 0.05. Correlation coefficients were determined using the Pearson product-moment correlation coefficient. Significance of correlation was defined if *p*-values < 0.05 were obtained. All statistical analyses were performed with SPSS^®^ 21.0 software (SPSS, Chicago, IL, USA). The Bonferroni post-hoc test was implemented for data comparison purposes. 

## 3. Results

**Figure 1:**
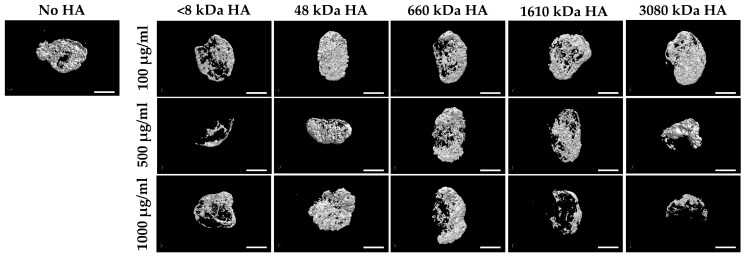
Micro CT images of BMP-2/ACS constructs (10 µg BMP-2 per sample) in the presence or absence of 100, 500, or 1000 µg/mL HA with different polymer sizes (<8, 48, 660, 1610, 3080 (kDa)) at 18 days after implant placement (Bar = 2.5 mm).

The screening experiments revealed that the HA polymer of 48 kDa molecular weight was able to yield the highest osteogenesis activity, when applied at a concentration of 100 µg/mL (dosage volume: 20 µL) of HA (Figure 1), and with an added BMP-2 amount of 10 µg (BMP-2 concentration in the solution: 1 µg/µL; BMP-solution-volume added: 10 µL/sample). 

**Figure 2:**
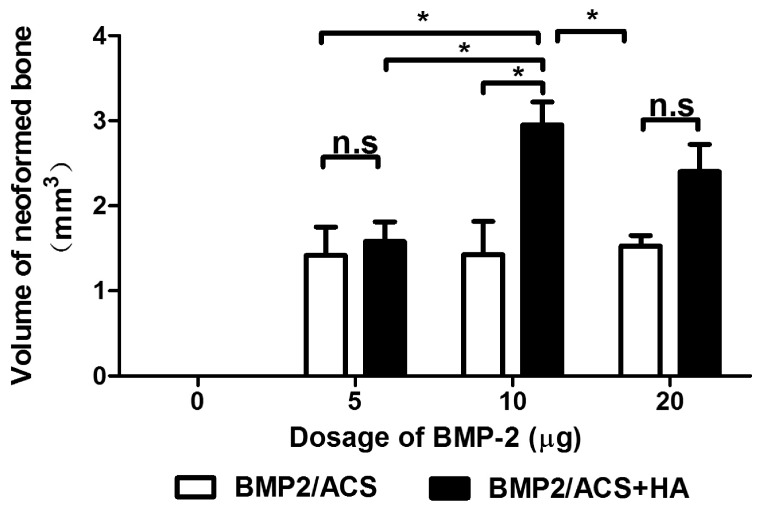
Mean volumes of newly formed bone tissue in the BMP-2/ACS constructs, in the presence or absence of 100 µg/mL HA (48 kDa) implanted at different BMP-2 dosages, 18 days after implant placement. Values represent means ± SEM; *n* = 6 per experimental group. The asterisks denote the level of statistical significance, i.e., * *p* < 0.05. The compared groups are indicated by brackets.

5, 10 and 20 μg BMP-2 resulted in a similar total volume of newly formed bone tissue, while no bone was detected with or without HA in the absence of BMP-2 (Figure 2). The combined administration of HA significantly increased the volume of neoformed bone in the 10 µg BMP-2 group (*p* = 0.024) by approximately 100%. HA also increased new bone formation in the 20 µg BMP-2 group, which was, however, insignificant (*p* = 0.3). In the 5 µg BMP-2 group no such enhancement effect was observed.

**Figure 3:**
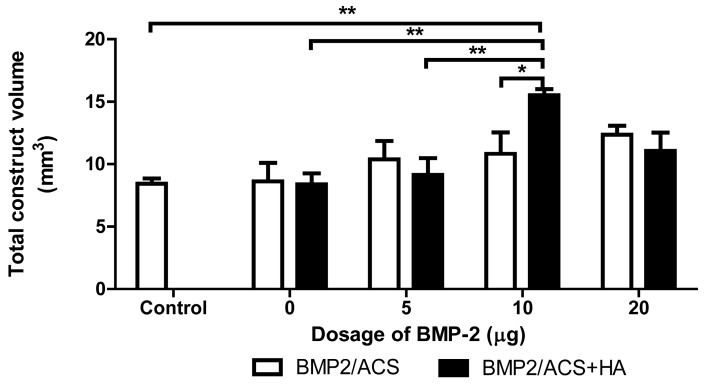
Mean volumes of total construct volumes of the BMP-2/ACS constructs, in the presence or absence of 100 µg/mL HA (48 kDa) implanted at different BMP-2 dosages; 18 days after implant placement. Values represent means ± SEM; *n* = 6 per experimental group. The asterisks denote the level of statistical significance, i.e., * *p* < 0.05, ** *p* < 0.01, *** *p* < 0.001). The compared groups are indicated by brackets.

The total construct volumes did not significantly differ among the groups without HA (Figure 3). However, among the groups with HA, the total construct volume of the 10 µg BMP-2 group in the presence of HA showed a significantly higher volume than the 5 µg BMP-2 group (*p* = 0.03) and 0 µg BMP-2 group (*p* = 0.007), respectively, but not the 20 µg BMP-2 group. Only the 10 µg BMP-2 group with HA resulted in a significantly higher total construct volume when compared to the time 0 (control group).

**Figure 4:**
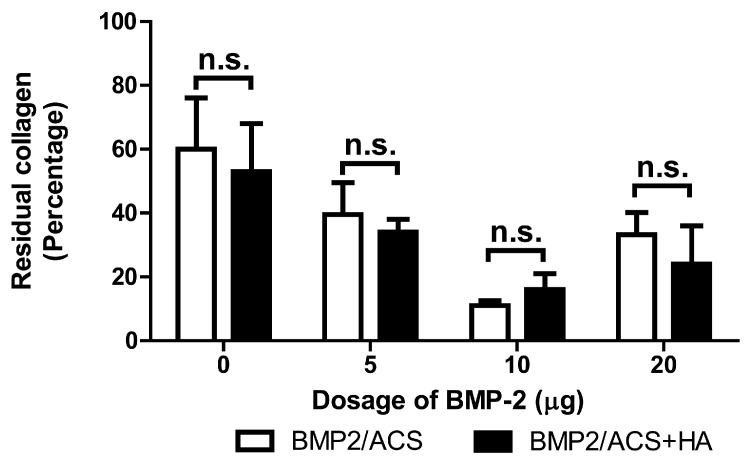
Mean volumes of residual collagen carrier material of the BMP-2/ACS constructs, in the presence or absence of 100 µg/mL HA (48 kDa) implanted at different BMP-2 dosages, 18 days after implant placement. Values represent means ± SEM; *n* = 6 per experimental group. n.s.: denotes the absence of significant differences (*p* > 0.05). The compared groups are indicated by brackets.

The volumes of remaining ACS showed a decreasing trend from the 0 µg BMP-2 group to the 10 µg BMP-2 group; the trend then reversed to the 20 µg BMP-2 group (Figure 4). Computation of the coefficient of correlation between the first three dosages (0, 5, and 10 µg BMP-2) in the absence of HA revealed *a* value for *r* = −0.62 (*p* = 0.006), i.e., a significantly correlated trend was present; in the presence of HA and the same BMP-dosage groups, the correlation coefficient was *r* = −0.459 (*p* = 0.075). The combined administration of HA did not significantly influence remaining ACS volumes for each dosage group. The coefficients of variations (CV) and coefficients of errors (CE) varied between CV = 69% (CE = 35%) for the 0 µg BMP group with HA, and CV = 27.8% (CE = 13.9%) for the 10 µg BMP group without HA.

**Figure 5:**
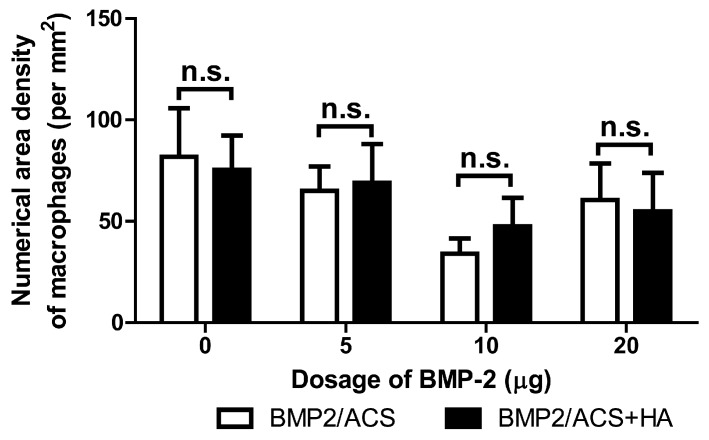
Mean values of the numerical area densities of macrophage cell profiles in the BMP-2/ACS constructs, in the presence or absence of 100 µg/mL HA (48 kDa) implanted at different BMP-2 dosages, 18 days after implant placement. Values represent means ± SEM; *n* = 6 per experimental group. n.s.: denotes absence of significant differences (*p* > 0.05). The compared groups are indicated by brackets.

**Figure 6:**
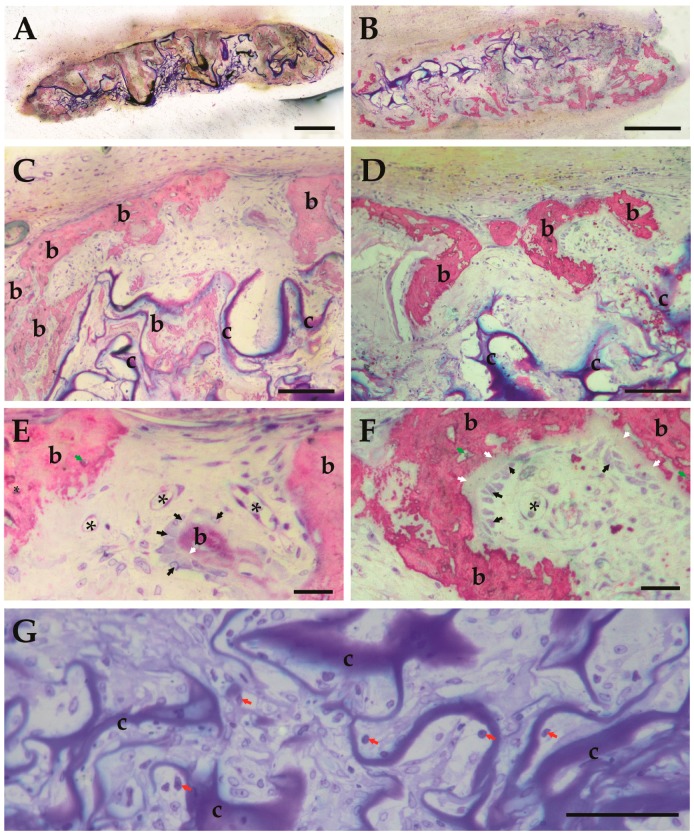
Light micrographs of BMP-2/ACS constructs, in the presence or absence of 100 μg/mL HA (48 kDa) at time of retrieval (18 days) at low (**A**,**B**) and high (**C**–**G**) magnifications: (**A**,**C**,**E**,**G**): BMP-2 10 µg+HA; (**B**,**D**,**F**): BMP-2 10 µg in the absence of HA. (**A**) illustrates homogenous bone forming activities throughout the construct, whereas in B formation of new bone tissue occurs preferentially at the interface of the construct with the native tissue. (**C**,**D**) illustrate the newly-formed bone tissue (b) in these two groups at higher magnifications and remaining collagen carrier material (c). (**E**,**F**) illustrate the blood vessels (*) and unmineralized bone areas (white arrow); osteoblasts (black arrow). In (**E**,**F**), the newly-formed woven bone shows a typical irregular pattern of osteocyte distribution (green arrow) within the mineralized bone matrix (pink-red stained areas). In (**E**) larger numbers of blood vessels (*) are present compared to (**D**); (**G**) illustrates the macrophages (red arrow) within BMP-2/ACS constructs. Magnification bars in (**A**,**C**): 500 μm; in (**C**,**D**,**G**): 100 μm; in (**E**,**F**): 25 μm.

**Figure 7:**
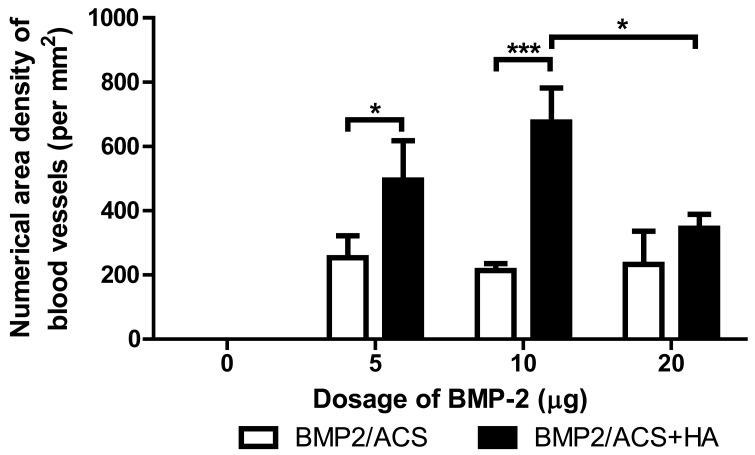
Mean values of the numerical area densities of blood vessel profiles in the BMP-2/ACS constructs, in the presence or absence of 100 µg/mL HA (48 kDa) implanted at different BMP-2 dosages, 18 days after implant placement. Values represent means ± SEM; *n* = 6 per experimental group. The asterisks denote the level of statistical significance, i.e., (* *p* < 0.05, ** *p* < 0.01, *** *p* < 0.001). The compared groups are indicated by brackets.

No significant differences in numerical area density of macrophages were present among these groups (Figure 5, Figure 6G). The 10 μg BMP-2 group value also was found to be significantly higher than the number of cross-sectioned blood vessels per unit tissue area in the 20 µg BMP-2 exerimental group (*p* = 0.02); but it did not significantly differ compared to the group of 5 µg BMP-2+HA (Figure 7). The combined administration of HA significantly promoted the the number of blood vessel in the 5 μg (*p* = 0.017) and 10 µg BMP-2 dosage groups (*p* = 0.0001), but not in the 20 μg BMP-2 group.

In the 10 µg BMP-2+HA group (Figure 6A,C), significantly less ACS and larger volumes of new bone were present when compared to the 10 µg BMP-2 group (Figure 6B,D). The number of cross-sectioned blood verssels was higher in Figure 6E than in Figure 6F, and that in Figure 6E the cross-section areas of the blood vessels are generally smaller. The computation of the average blood vessel cross-sectioned area, obtained by dividing the mean blood vessel areal density by the mean number of blood vessel cross-sections per area, revealed that the mean area per vessel for the 10 µg BMP-2 +HA group is 0.7 × 10^−4^ mm^2^, and the mean area per blood vessel for the 10 µg BMP-2 group without HA is 2 × 10^−4^ mm^2^; thus, the mean cross-sectioned blood vessel area is about three times larger in the experimental group in the absence of HA than in the same BMP dosage group in the presence of HA. In addition, histological observation revealed that in the 10 µg BMP-2 group without HA, the typically observed patterns of carrier degradation and new bone formation differed: whereas bone formation activities generally occured throµghout the ACS carrier materials (see Figure 6A), in the 10 μg BMP group in the absence of HA the new bone formation activities occured preferentially in the peripheral areas of the carrier materials (Figure 6B). However, the qualitiy of newly-formed bone tissue was found upon morphological examination to be the same in all experimental groups; in particular, the numerical density of osteoclasts appeared to be the same in all groups in which bone tissue had been generated, and no decline or change of the osteoclast numerical density was observed in any experimental group, in particular not in the 10 μg BMP group+HA group. 

## 4. Discussion

HA is one of the major physiological components of the extracellular matrix (ECM), in all the connective tissues of the body. It is involved in a number of major biological processes, such as tissue organization, wound healing, angiogenesis, and remodeling of skeletal tissues [36,37,38]. In addition, HA is polyanionic in nature and, therefore, capable of forming ionic bonds with cationic growth factors, such as BMPs, which seems to be of significance for clinical applications [38]. In this study, we found that the combined administration of HA could significantly enhance the osteogenic potential of BMP-2/ACS, allowing a minimized unwanted side-effects [7].

Our extensive preliminary screening experiments revealed that an HA polymer length of about 48 kDa was of the optimal size range for the desired effect when used at a concentration of approximately 100 µg/mL. This might be because HA established, at these conditions, the optimal form of a gel, in which BMP-2 was most efficiently entrapped to optimally retain its bioactivity [39]. As a meshwork, HA might also reduce the free diffusion capabilites of BMP-2 and its flow, thus acting as a slow release system with an enhanced osteogenic activity potential [40].

In the present study, HA, at the optimal specifications, clearly promoted the BMP-dependent osteogenesis activity (Figure 2). In addition, the total carrier volume (Figure 3) and the number of blood vessel cross-sections per unit area of tissue, were also the highest in the 10 μg BMP group+HA group (Figure 6). Such effects were indeed absent in all other experimental groups without HA where the generated new bone mass did not even vary as a function of different BMP-2 dosage levels (Figure 2). The promoting effect of HA on new bone formation was only seen at dosages higher than the 10 μg BMP group (Figure 2), which suggested that this group might thus lie in the range of a minimal BMP dosage needed for the desired effect of higher bone volume generation in the present conditions. 

The inflammatory response to BMP-2/ACS, was found to be the same in all experimental groups (Figure 5). The HA-dependent promoting effect for bone formation was unlikely attributed to a modification effect of HA on the inflammatory response. Instead, the HA-dependent facilitating effect on bone formation might be more likely associated with the degree of formation of new blood vessels, i.e., with the angiogenetic activity associated with the osteogenetic response. On one hand, we found that the number of cross-sectioned blood vessels was clearly the highest in the 10 μg BMP-2 group; on the other hand, this effect is clearly associated with the presence of a higher total surface area of blood vessel walls and, thus, of a larger blood vessel-wall associated perivascular tissue space, than when only fewer and thicker blood vessels are present; and it is indeed the peri-vascular tissue area that is the niche space carrying the pericytes and, thus, harbors the population of blood vessel associated adult stem cells of the mesenchymal type [41]; these have been previously found and identified to be able to differentiate into bone forming osteoblasts [42]. 

HA polymers showed an angiogenetic effect at specific polymer lengths [43], and BMP-2 itself was also shown to have itself some angiogenetic activity [44]. In addition, HA could also facilitate the migration of the perivascular stem cells [45] from their original niche to distant sites within the newly-forming tissues. HA is well-known to stimulate signal transduction pathways [46,47] that in turn facilitate cell locomotion [47]. Moreover, our data were also consistent with a recent study of Jungju Kim [48]: he found that BMP-2 activity was accompagnied only with the highest expression of osteocalcin and with a mature form of bone tissue with positive vascular markers (such as CD31 and vascular endothelial growth factors) when applied in the presence of HA, illustrating again that acitive angiogenesis was one of the key factors accounting for successful new bone formation [49].

It should always be kept in mind that BMP-activity is also associated with the recruitment, formation, and activation of osteoclasts, leading to immediate bone resorption activities. In this study no significant variation of osteoclast density in the newly formed bone tissue compartments among the groups. Thus, it appears unlikely that a lower degree of bone resorption activity would be a significant factor in supporting the formation of higher bone volumes in the 10 μg BMP group. It was, indeed, the careful dosage that was needed for BMP-2 in order to work out the required balanced-dosage of minimizing the osteoclastogeneic effects of BMP-2 and maximizing the osteogenetic effects of this pleiomorphic growth factor as we recently illustrated in sheep [40]. 

The clearly higher degree of blood vessel numbers and, thus, blood vessel wall surface area in the 10 µg BMP-2 group highly suggested that the HA-dependent osteogenic promotion effect of BMP-2 was related to a concomitantly associated increased angiogenetic activity. The fact that the total construct volume was also the largest one for the 10 μg BMP-2 group among all the experimental groups, supported this view since this large total construct volume was mainly due to the increased presence of bone tissue, and not to an increased volume of inflammatory area or swelling effect; moreover the volume of the residual ACS was indeed the smallest one in this group, both in relative (Figure 4) and absolute terms (data not shown). The high degree of scatter of the mean values of the residual collagen in the experimental groups, represented by the coefficients of variations of these groups, was, however, fairly large, and again it was the smallest for the 10 μg BMP-2 groups (Figure 4); the CE of the 10 μg BMP-2 group in the absence of HA was 13.9%, and in the presence of HA was 30.6%. We were, thus, unable to put forward a clear explanation for our finding, but we are inclined to assume that this result is associated with a more rapid and efficient degradation of the collagen carrier materials deposited. However, since the degree of inflammatory response was quite similar in all groups (Figure 5), and no significant differences were encountered, it could be speculated that this phenomenon might be associated with a higher degree of osteolytic activity in this group; i.e., with a more rapid bone resorption activity in this group with the highest bone mass. There were, however, no indications found for the presence of higher numbers of osteoclasts in this group, and indeed the detailed morphological examination did not reveal any differences between groups in this respect. However, another possible (and more likely) explanation may be related to the more extensive angiogenetic activity encountered in this group: rapidly ingrowth and forming new blood vessels may be associated with the more efficient degradation of the collagen carrier materials, and indeed angiogenesis associated with tissue engineering approaches was previously described to be associated with such degradative activities [50]. Another indicator for favoring this hypothesis was the specific morphological pattern of new bone formation observed in this group: whereas, in all the other experimental groups, new bone tissue had formed mainly at the periphery of the constructs where probably most blood vessels were present, i.e., at the interface of the vascularized native tissue with the avascular construct (and bone tissue indeed does not form in the absence of a blood vasculature [51]). This pattern of bone formation relating to an osteogenic construct using ACS as carrier was observed by us also in a recent study [15]. However, the 10 μg BMP-2 group is the only one in which bone formation activities occurred by a different pattern, namely throughout the carrier construct with blood vessels being present all the way through the construct at high numerical densities (Figure 7). It appeared more probable that the more efficient degradation activities for the ACS (Figure 4) were associated with this more aggressive angiogenetic activity. 

## Figures and Tables

**Table 1:** Screening parameters.

HA-Moleculer Weights (kDa)	HA-Concentrations (µg/mL)	BMP-2-Dosages (µg)
<8	50	0
48	100	5
660	500	10
1610	1000	20
3100		
